# Camera-based optical palpation

**DOI:** 10.1038/s41598-020-72603-5

**Published:** 2020-09-29

**Authors:** Rowan W. Sanderson, Qi Fang, Andrea Curatolo, Wayne Adams, Devina D. Lakhiani, Hina M. Ismail, Ken Y. Foo, Benjamin F. Dessauvagie, Bruce Latham, Chris Yeomans, Christobel M. Saunders, Brendan F. Kennedy

**Affiliations:** 1 BRITElab, Harry Perkins Institute of Medical Research, QEII Medical Centre, Nedlands and Centre for Medical Research, The University of Western Australia, Perth, WA 6009 Australia; 2 Department of Electrical, Electronic & Computer Engineering, School of Engineering, The University of Western Australia, Crawley, WA 6009 Australia; 3 PathWest, Fiona Stanley Hospital, 11 Robin Warren Drive, Murdoch, WA 6150 Australia; 4 Division of Pathology and Laboratory Medicine, Medical School, The University of Western Australia, Crawley, WA 6009 Australia; 5 The University of Notre Dame, Fremantle, WA 6160 Australia; 6 Division of Surgery, Medical School, The University of Western Australia, Crawley, WA 6009 Australia; 7 Breast Centre, Fiona Stanley Hospital, 11 Robin Warren Drive, Murdoch, WA 6150 Australia; 8 Breast Clinic, Royal Perth Hospital, 197 Wellington Street, Perth, WA 6000 Australia; 9 Australian Research Council Centre for Personalised Therapeutics Technologies, Melbourne, Australia; 10 Present Address: Visual Optics and Biophotonics Group, Instituto de Óptica “Daza de Valdés”, Consejo Superior de Investigaciones Científicas (IO, CSIC), C/Serrano, 121, Madrid, Spain

**Keywords:** Biomedical engineering, Imaging and sensing

## Abstract

Optical elastography is undergoing extensive development as an imaging tool to map mechanical contrast in tissue. Here, we present a new platform for optical elastography by generating sub-millimetre-scale mechanical contrast from a simple digital camera. This cost-effective, compact and easy-to-implement approach opens the possibility to greatly expand applications of optical elastography both within and beyond the field of medical imaging. Camera-based optical palpation (CBOP) utilises a digital camera to acquire photographs that quantify the light intensity transmitted through a silicone layer comprising a dense distribution of micro-pores (diameter, 30–100 µm). As the transmission of light through the micro-pores increases with compression, we deduce strain in the layer directly from intensity in the digital photograph. By pre-characterising the relationship between stress and strain of the layer, the measured strain map can be converted to an optical palpogram, a map of stress that visualises mechanical contrast in the sample. We demonstrate a spatial resolution as high as 290 µm in CBOP, comparable to that achieved using an optical coherence tomography-based implementation of optical palpation. In this paper, we describe the fabrication of the micro-porous layer and present experimental results from structured phantoms containing stiff inclusions as small as 0.5 × 0.5 × 1 mm. In each case, we demonstrate high contrast between the inclusion and the base material and validate both the contrast and spatial resolution achieved using finite element modelling. By performing CBOP on freshly excised human breast tissue, we demonstrate the capability to delineate tumour from surrounding benign tissue.

## Introduction

Optical elastography describes a range of techniques used to image the mechanical properties of biological tissue on the micro- to millimetre scale^1–5^. The main application of these techniques is in medical imaging as it is well-established that there is often a correlation between disease and tissue mechanical properties on these scales^1,6–10^. The use of optics invariably provides higher spatial resolution than alternative approaches, namely, ultrasound elastography^11^ and magnetic resonance elastography^12^, and affords the opportunity to implement small form factor probes which are amenable to in vivo imaging^13–15^. A range of approaches have been developed that each utilise an optical imaging modality to measure deformation induced by a mechanical load. A mechanical model of the tissue deformation is then used to estimate a mechanical property or parameter that is mapped into an image. The most widely developed approach is optical coherence elastography (OCE)^2,3,16–19^ which combines optical coherence tomography (OCT) and a loading mechanism, typically either a quasi-static compression or an acoustic excitation, to measure the local mechanical response of tissue from which a map of elasticity is derived. OCE has an attractive combination of features, including rapid acquisition, micrometre-scale resolution and millimetre to centimetre field-of-view^20,21^, and has been proposed for a number of applications including tumour margin assessment and keratoconus detection^22–26^. Brillouin microscopy is another prominent technique which utilises confocal microscopy to measure the Brillouin frequency shift induced by vibrations of phonons in the tissue. The local longitudinal (bulk) modulus of the sample is derived from this frequency shift and is related to elasticity under simplifying assumptions^27–29^. Brillouin microscopy has been demonstrated in cell mechanics^29,30^ and ophthalmology^31,32^, where particular advantages include its non-contact configuration and the fact that no external loading mechanism is required.

Whilst these existing high resolution variants of optical elastography offer capabilities to image at, or close to, the cellular scale, to imaging depths of hundreds of micrometres to several millimetres, they typically rely on expensive imaging systems that restrict application of optical elastography to niche areas, such as tumour margin assessment^22^. Furthermore, existing techniques may not be practical in low-resource and remote settings^33–35^. Another challenge is that techniques such as OCE and Brillouin microscopy are not easily used by non-optics experts as they contain complex optical components that require careful alignment, thus precluding a broader application of the technology. Also, in some applications, it would be advantageous to have a device with a small footprint and without the burden of optical patch cords obstructing the practical use of the device. For example, there is great promise for optical elastography in the food industry where the elasticity of meats and fruits is an important indicator of food quality^36^ and in the robotics industry where tactile feedback is of vital importance^37^. However, these applications remain largely out of reach of existing optical elastography techniques.

In this paper, we begin to address this gap in optical elastography capabilities by proposing camera-based optical palpation (CBOP), a novel approach which provides stress maps, termed optical palpograms, at the tissue surface using a relatively low-cost (< $1,000 USD), 12.2 megapixel (MP), complementary metal-oxide semiconductor (CMOS) camera and a micro-porous compliant silicone layer. Analogously to the sense of touch, the stress measured in optical palpograms is dependent on the stiffness of the underlying tissue. In CBOP, the light intensity transmitted through the micro-porous layer placed on top of the tissue of interest is detected. The refractive index difference between the silicone and the air inside the micro-pores causes light scattering, such that minimal light is transmitted through the layer in its uncompressed state. However, when compression is applied to the layer, the micro-pore volume decreases leading to an increased transmission of light. In this way, the camera intensity encodes the strain of the layer. Pre-characterisation of the relationship between stress and strain in the layer using standard compression testing allows for optical palpograms to be derived from the measured strain, endowing CBOP with the capability to generate mechanical contrast from a simple digital camera. Another advantage of CBOP is that the images generated are independent of the optical properties of the underlying tissue, as only the light transmitted through the layer is required to generate mechanical contrast. This may provide a benefit over techniques such as OCE and Brillouin microscopy where the optical scattering from the tissue is often inconsistent and can be obscured, for example, by the presence of blood.

CBOP is inspired by OCT-based optical palpation^38^, where OCT intensity is used to measure the thickness of a pre-characterised non-porous, transparent silicone layer compressed against the tissue. The strain in the layer is determined by the thickness change measured from the OCT data and the known stress–strain relationship is then used to generate optical palpograms. OCT-based optical palpation has been demonstrated to provide high imaging contrast for skin and human breast tissue^39,40^. For example, in one study of excised human breast tissue from 34 patients, strong correlation was observed between optical palpograms and corresponding gold standard histology^40^. However, a main drawback of OCT-based optical palpation is the requirement to use an expensive optical imaging system. In addition, a 3-D OCT data set is required, typically restricting the acquisition time needed to generate an optical palpogram to the range of seconds^41^, and furthermore, placing a substantial burden on signal processing, limiting its application in scenarios where rapid visualisation of palpograms is essential.

In this paper, we first describe the fabrication and characterisation of the micro-porous silicone layer. We then compare the performance of our technique to OCT-based optical palpation on four structured silicone phantoms, containing stiff inclusions ranging from 0.5–5 mm in size, by analysing the contrast-to-noise ratio (CNR) and spatial resolution in each case. This analysis is accompanied by validation of the mechanical contrast using finite element modelling (FEM). Finally, we demonstrate our technique on human breast specimens freshly excised from mastectomy surgeries. These tissue results are co-registered with and validated against post-operative histology, verifying that CBOP can distinguish tumour from surrounding benign tissue.

## Materials and methods

### Fabrication of the micro-porous layer

**Figure 1:**
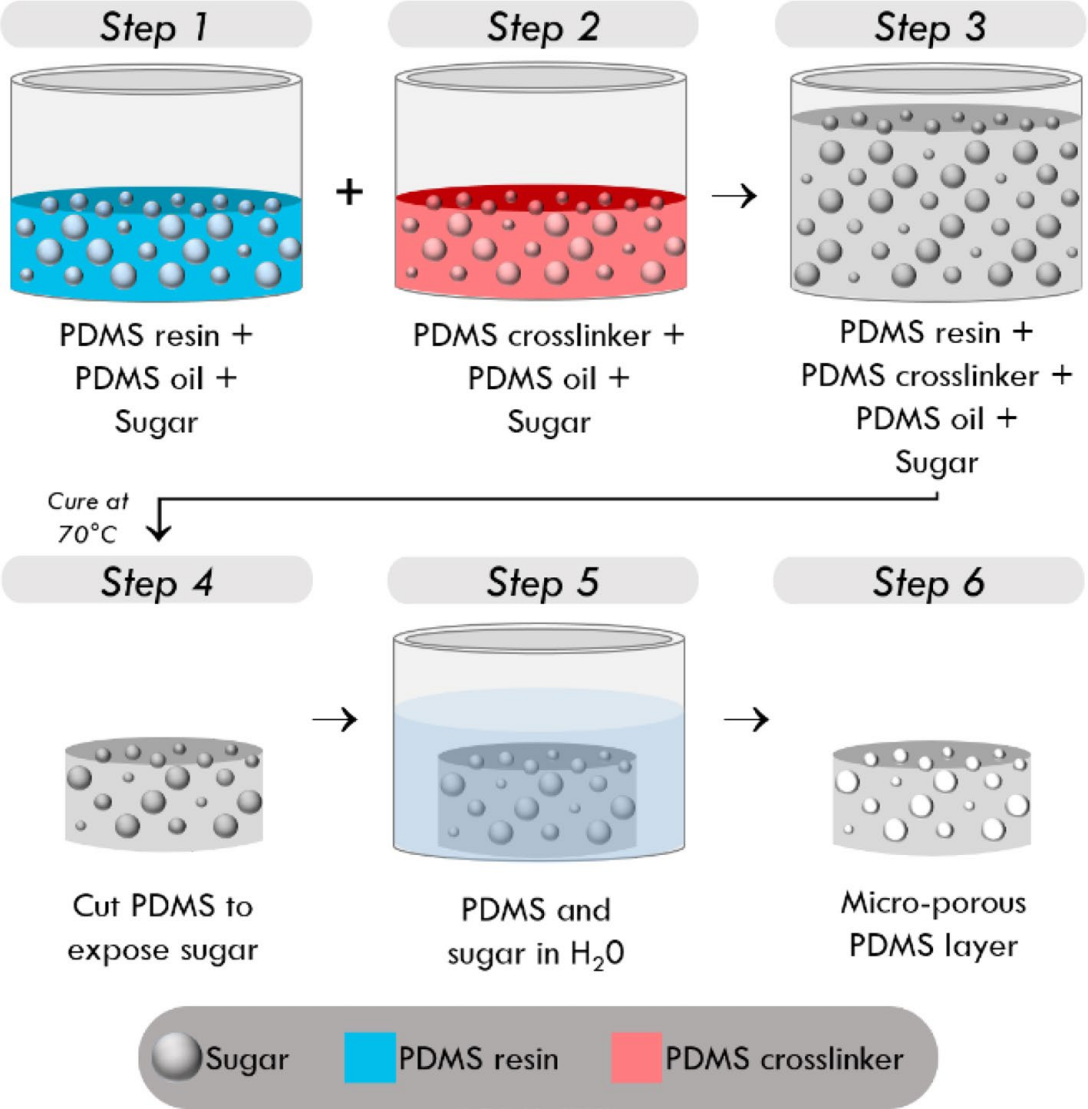
Fabrication process of micro-porous silicone layers by sacrificial templating of sugar grains.

The micro-porous layers used in CBOP consist of a polydimethylsiloxane (PDMS) matrix and an open cell network of ~ 30–100 µm diameter pores. While there are several methods for porous layer fabrication, such as the embedding of hollow micro-spheres into the pre-cured base material^42^ and gas foaming^43^, in CBOP a direct templating technique^44,45^ is employed, which involves mixing a sacrificial substrate (sucrose) into the PDMS prior to curing. The sugar is dissolved out after curing, resulting in an open cell network. The layers are cut into 25 mm diameter discs, ~ 900 µm thick, and exhibit an elasticity of 7.3 kPa at 10% strain. The steps in this process are illustrated in Fig. 1, and are described in detail below:

Step 1: PDMS resin (Elastosil P7676 Part A, Wacker Chemie, Munich, Germany) and PDMS oil (Wacker AK 50, Wacker Chemie, Munich, Germany) are first mixed at a ratio of 2:1. Adding oil to the resin reduces the overall elasticity of the cured PDMS matrix^46^. Then, fine grain sugar (grain size ~ 30–100 µm) is added to the compound in a concentration of 1 g/ml. Grain size uniformity is ensured by passing the sugar grains through both a 106 µm sieve (Product: 003SAW.106, Endecotts, London, United Kingdom) and a 32 µm sieve (Product: 003SAW.032, Endecotts, London, United Kingdom) and collecting the sugar that did not pass through the smaller sieve. Using fine grain sugar allows for uniform dispersion of the grains throughout the matrix, as the suspension is less likely to sediment in the PDMS^47^. This compound is stirred for 5 min using a propeller stirrer, to allow even distribution of the sugar particles.

Step 2: PDMS crosslinker (Elastosil P7676 Part B, Wacker Chemie, Munich, Germany) is mixed with the same type of PDMS oil used in Step 1 at a mixing ratio of 2:1. Then, sugar with the same grain size as in Step 1 is added to the compound at a concentration of 1 g/ml, followed by thorough stirring for 5 min to allow even distribution of the sugar particles in the compound. Steps 1 and 2 are performed separately due to the high viscosity of the mixture making it difficult to combine the sugar and PDMS uniformly in the timeframe before the PDMS begins to cure.

Step 3: The resin and crosslinker compounds are combined with additional PDMS oil at a ratio of 1:1:1 and stirred for 5 min to provide complete mixing. Then, a portion of the mixture is pipetted into a petri dish, such that the total thickness is 900 µm. The mixture is then placed in an oven at 70 °C for 30 min, to provide temperature-accelerated curing.

Step 4: After curing, the mixture is cut into cylinders (diameter, 25 mm) using a blade and a circular punch. The cut surfaces of the cylindrical layer provide exposure of the sugar particles to air, which is important for the dissolution process in the next step.

Step 5: The cylindrical layer is placed in a water bath at room temperature, to allow dissolution of the sugar particles. It takes up to 72 h to fully dissolve the sugar particles, due to the small diameter of the inter-connected channels between sugar particles. To accelerate the dissolution process, the water bath can be kept at 70 °C in the oven, increasing the thermal energy used to break intermolecular bonds between sucrose molecules. Using this method, the total period of dissolution can be reduced to 48 h.

Step 6: The layer with the sugar particles fully dissolved is removed from the water bath, followed by a process of dehydration to remove any residual water inside the layer. This involves placing the layer in the oven at 70 °C for 48 h to remove any residual moisture. After this, the micro-porous layer is ready to be used in imaging.

### Experimental design

CBOP uses a 12.2 MP CMOS camera (Basler ace acA4024-29uc, Basler AG, Ahrensburg, Germany) and a 25 mm fixed focal length lens with a working distance of 100 mm, costing $550 USD and $250 USD, respectively. The camera and lens are positioned above a rigid glass window, dimensions: 50.8 × 50.8 × 3 mm, that acts as a compression plate (high efficiency window, Edmund Optics, New Jersey, USA). During acquisition, the sample, a 900 µm-thick micro-porous layer and a ~ 300 µm-thick green layer are placed on a rigid plate which is affixed to a translation stage (MTS25-Z8, Thorlabs, New Jersey, USA). The green layer, made from mixing one part of green silicone-based pigment (SP-Green, Barnes Products, Moorebank, Australia) with four parts silicone elastomer (Elastosil P7676, Wacker Chemie, Munich, Germany) serves as a homogeneous colour mask for the underlying sample and ensures that variability in light backscattered from the sample does not affect the estimation of stress. The layer is dyed green as the CMOS camera exhibits a higher spectral sensitivity to this colour channel. 200 µL of PDMS oil (Wacker AK 50, Wacker Chemie, Munich, Germany) is applied between each mechanical interface to reduce the effect of friction which restricts the lateral expansion of the sample, the green layer and the micro-porous layer under compression. The translation stage is operated in the *z*-direction such that the stage compresses the sample and layers against the glass window.

During the experiment, the translation stage is used to bring the sample into contact with the glass window, at which point the total thickness of the sample together with the green layer and micro-porous layer is recorded. The initial thicknesses of the green layer and micro-porous layer are also measured prior to the experiment. Then, the translation stage is used to increase the preloaded strain applied to the overall thickness (sample + green layer + micro-porous layer) in 10% increments until 50% preloaded strain is reached. At each of the strain levels, ten digital photographs are acquired at a rate of 10 fps and are later averaged in post-processing to reduce the effect of shot noise on optical palpograms. Averaging multiple photos at a preloaded strain improves the SNR by as much as 30%, at the expense of longer acquisition times. After imaging, the green and micro-porous layers are removed, and the sample is loaded to the same approximate strain to acquire a digital photograph of the tissue surface to assist with co-registration of features in the optical palpogram.

**Figure 2:**
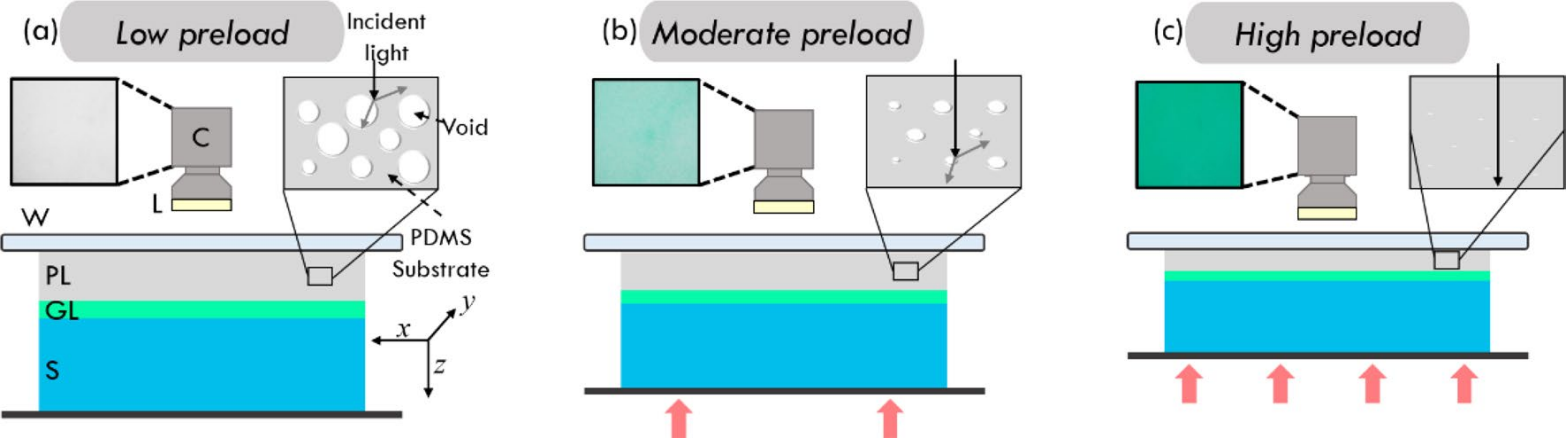
Working principle of CBOP. A CMOS camera is used to measure the transmission of light emitted by LEDs through the micro-porous layer and reflected back from the green layer under (**a**) low preloaded strain, (**b**) moderate preloaded strain and (**c**) high preloaded strain; *C* camera, *L* LEDs, *W* glass window, *PL* micro-porous layer, *GL* green layer, *S* sample. The insets from the camera show the change in green intensity with different preloaded strains. The micro-porous layer inset illustrates the reduction in pore size under increasing compression.

Figure 2 shows a schematic of the experimental setup. In Fig. 2a, at a low level of strain, the light emitted by an array of 6,000–6,500 K SMD 5050 white-light LEDs situated above the imaging window, reaches the micro-porous layer and the air-filled pores scatter light and prevent transmission to the green layer below, resulting in the camera detecting white light as shown by the inset in Fig. 2a. The LEDs in the array each emit ~ 20 lm and draw 60 mA at 3 V. Additionally, they are housed in a 3D printed plastic case which blocks out the ambient light, preventing any light in the surrounding environment from affecting the acquired image. As the compression is increased (Fig. 2b), the pores become smaller and partially close, allowing a portion of the light to transmit through to the green layer, resulting in the detection of green light on the CMOS sensor. When the compression is increased further, as shown in Fig. 2c, the level of green light detected by the CMOS sensor increases. Characterisation of the relationship between the light transmission and the stress of the micro-porous layer is presented in the next section.

### Optical transmission through the micro-porous layer

Light travelling through the micro-porous layer undergoes refraction, due to the difference in refractive indices, *n*, between the silicone matrix (*n* = 1.4) and the pores (*n* = 1.0)^46^. This refraction causes light to scatter multiple times in the layer, reducing optical transmission. Optical transmission of light *T*, in the micro-porous layer is proportional to the distance a photon will travel before its direction is randomised after scattering by a pore, termed the transport mean free path *l*_*mfp*_*,* and the reciprocal of the total thickness of the material, *L*, assuming negligible absorption^48^. Mie scattering theory describes scattering interactions where the pore size is comparable to or greater than the wavelength of light. It suggests that the transport mean free path is inversely proportional to both the number of pores in a given volume, termed, the pore concentration, *φ*, and the pore scattering cross-section^49,50^, defined as the effective area proportional to the probability of an incoming photon interacting with the pore. Provided the pore is non-absorbing and much larger than the wavelength of the incident light, the scattering cross-section is linearly proportional to the pore cross-sectional area, *A*^51,52^. The micro-porous layers used in CBOP satisfy the material thickness and pore size conditions of Mie scattering theory, and therefore, the optical transmission can be approximated as inversely proportional to the thickness, pore concentration and pore cross-sectional area.

1 $$T\propto \frac{1}{L\varphi A}$$

**Figure 3:**
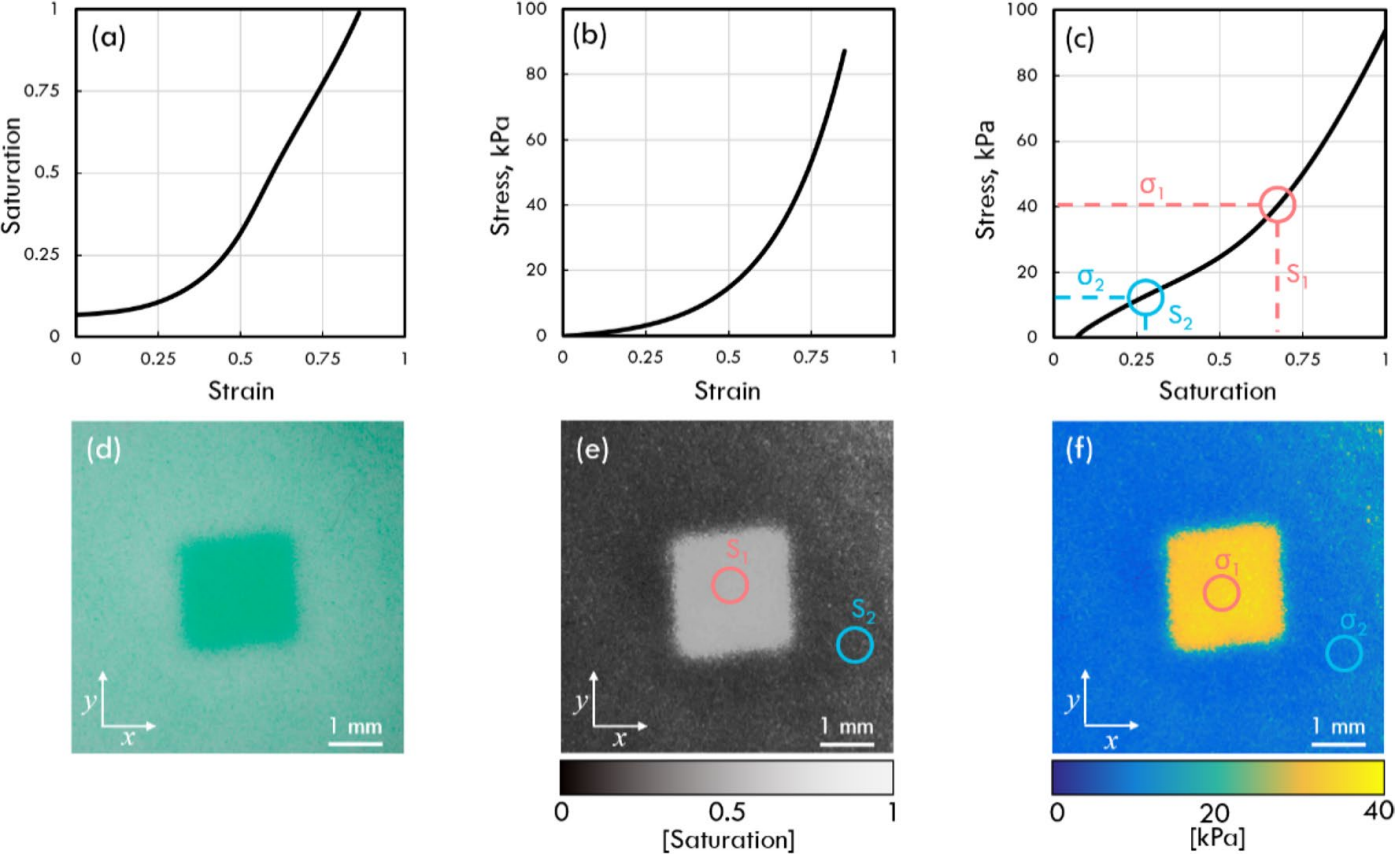
Layer characterisation and generation of optical palpograms. (**a**) The saturation-strain characterisation curve, (**b**) the stress–strain characterisation curve of the micro-porous silicone layer and (**c**) the resulting stress-saturation curve. (**d**) Digital photograph of micro-porous layer of phantom containing a 2.5 × 2.5 mm inclusion phantom at 50% preloaded strain, and (**e**) the corresponding colour saturation image where the red and blue circles represent the relative colour saturation through a region of the inclusion and base, respectively. (**f**) The optical palpogram is produced by equating each pixel in (**e**) to a stress value using the stress–strain curve in (**c**).

The above relationship describes the transmission of light through the micro-porous layer under static conditions. However, in CBOP, the optical transmission is dependent on the applied strain. Increasing compressive loading on the micro-porous layer not only reduces the thickness of the material^53^, but also causes the internal pores to collapse as the silicone matrix expands into the voids^54^, reducing the total pore concentration of the layer, in addition to reducing the geometrical cross-section of the pores. This is a result of the near incompressibility of silicone, which has a Poisson’s Ratio close to 0.5^55,56^. These changes to the pore structure, coupled with the reduction in material thickness, increase the transport mean free path, allowing more light to pass through the micro-porous layer. While compression also reduces the intrinsic backscattering of light from the pores, it affects all colour channels evenly and therefore has negligible impact on colour saturation. As a result, optical transmission can be directly related to the compression applied to the micro-porous layer, as shown in Fig. 3a.

### Post-processing

Post-processing of the acquired camera images is performed using MATLAB 2016B (MathWorks, Massachusetts, USA). Averaging of the ten acquired images at each preloaded strain is performed first, generating a 2-D pixel matrix with the average RGB values recorded at each pixel location as shown in Fig. 3d. Colour saturation is then used as a measure of optical transmission, as the micro-porous layer appears white (low saturation) at low preloaded strain and green (high saturation), at high preloaded strain. A 2-D saturation image is generated by measuring the saturation, *S*, from the RGB values at each pixel location using the following equation:

2 $$S=\frac{\alpha \left({C}_{max}-{C}_{{min}_{1}}\right)+\left({C}_{max}-{C}_{{min}_{2}}\right)}{\alpha {C}_{max}+{C}_{max}},$$

where *S* is the saturation value at each pixel.${C}_{max}$, ${C}_{{min}_{1}}$ and ${C}_{{min}_{2}}$ are the highest, lowest and second lowest of the RGB values at each pixel location, normalised between 0 and 1, and α is a user-defined coefficient used to optimise the contrast in the saturation image. This formula is a variation of the conventional colour saturation formula^57^ and was used to extend the stress dynamic range by considering RGB values from all three colour channels. In our experimental setup, optimised contrast is achieved with α = 1.5, as it provides a sufficiently large stress dynamic range (0–94 kPa), whilst providing minimal trade-off in sensitivity. Figure 3 shows the procedure for converting the averaged digital photograph (Fig. 3d) to the saturation image (Fig. 3e), then transforming the saturation image into an optical palpogram (Fig. 3f) for a silicone phantom with an embedded inclusion. First the colour saturation-strain curve of the micro-porous layer is characterised using the CMOS camera (Fig. 3a). Then, the stress–strain curve of the micro-porous layer is characterised using a uniaxial compression testing system (Fig. 3b). This system uses a motorized translation stage to increment the strain applied to a sample at a rate of 0.001/s. The strain is determined by measuring the initial thickness of the sample and the displacement of the stage. A load cell measures the applied force at a particular strain, which, along with prior knowledge of the geometric cross-section of the sample allows stress to be estimated. By dividing stress by strain, the elasticity can be estimated. By combining these two curves, we can generate a characteristic stress-saturation curve of the micro-porous layer, as shown in Fig. 3c.

### Layer characterisation and repeatability

The micro-porous layers are mechanically and optically characterised by connecting a load cell (LSB200, Futek Inc., California, USA) to the translation stage of the CBOP set-up. While compression is applied, the camera captures images of colour saturation as light is transmitted through the micro-porous layer and is reflected by the green layer, while the load cell records the resulting force, which along with prior knowledge of the layer surface area, is used to compute stress. To determine the repeatability of this procedure, characterisation is performed on five separate micro-porous layers. The resulting mechanical stress–strain curves demonstrate a mean standard deviation of 0.38 kPa across each of the measured preloaded strains. For the optical properties, the mean standard deviation in saturation values *S* is 0.03 across any given preloaded strain. This shows that there is a high level of repeatability in the fabrication process between individual layers.

### Finite element modelling

In order to validate the stress values measured from CBOP and to simulate the mechanical response of the micro-porous layer, 2-D FEM is performed on each inclusion phantom using Abaqus (Dassault Systèmes, Vélizy-Villacoublay, France). The FEM simulation is designed to match the experimental conditions in CBOP, where a stiff inclusion is embedded 500 µm below the surface of a soft silicone phantom. Two additional layers are positioned above the phantom, representing the green layer (300 µm thick) and the micro-porous layer (900 µm thick). The boundary conditions are fixed at the bottom of the phantom and 50% compression is applied axially via the imaging window, which acts as a rigid plate. The friction coefficient between all interfaces is set to 0.2 to account for the PDMS oil applied to the sample in the experiment and the 50% preloaded strain. This value was determined previously by Wijesinghe et al.^58^ by relating experimental results of OCT-based optical palpation to FEM simulations. 2‑D plane stress elements (CPS3) are assigned to all the models, which generate a maximum mesh size of 0.1 mm. Each element of the soft base, and both the green and micro-porous layers are modelled using the Mooney-Rivlin material model for uniaxial compression which relates stress to strain through the coefficients *C*_*10*_ and *C*_*01*_^59,60^:

3 $$\sigma = 2\left( {C_{10} + \frac{{C_{01} }}{\lambda }} \right)\left( {\lambda - \lambda^{ - 2} } \right),$$

where *σ* is the stress and $\lambda =1+\varepsilon$ and is defined as the stretch ratio. This model assumes that the materials are homogeneous, isotropic and non-linear. The Mooney-Rivlin coefficients for the soft silicone and green layer are given as C_10 _= 2.23 kPa and C_01 _= 0.70 kPa, respectively^58^. Similarly, the coefficients for the micro-porous layer are C_10 _= 0.04 kPa and C_01 _= 1.10 kPa. All coefficients are obtained by fitting Eq. 3 to the experimental stress–strain curves of each material which were generated through uniaxial compression testing. The relatively stiff inclusion, however, is modelled as an elastic material to increase the stability of the simulation. This is a valid assumption as the inclusion did not strain to more than 15% in each experiment and the stress–strain curve of the material is approximately linear in this range (elasticity of 160 kPa at 10% strain and Poisson’s ratio of 0.45). Stress is computed from the simulated strain using the same steps as in the CBOP experiments. The 2-D FEM is then expanded to 3-D for comparative analysis to the *en face* optical palpograms obtained using CBOP, as shown in “Results”. *En face* FEM images are imported into MATLAB for analysis and editing^61^.

### OCT-based optical palpation

OCT-based optical palpation is performed to provide a comparison to CBOP. This is achieved using a spectral-domain OCT system with central wavelength of 1,300 nm and spectral bandwidth of 170 nm (TEL320C1, Thorlabs, New Jersey, USA), in a common-path configuration^40^. An objective lens with a 0.055 numerical aperture (LSM03, Thorlabs, New Jersey, USA) is attached to the scan head which provides a measured full width at half maximum axial resolution of 5.5 µm (in air) and lateral resolution of 7.2 µm. A-scans are acquired at a rate of 71 kHz. The optical power on the sample was measured to be 2 mW. Prior to scanning, a 500 µm-thick homogeneous silicone compliant layer is placed on the sample (Elastosil P7676, Wacker Chemie, Munich, Germany), which ensures that the deforming edge of the layer is within the focus of the OCT system. The sample is then placed on a motorised lab jack to provide axial compression against a fixed glass window, situated between the sample and the OCT scanner. Whilst under compression, the OCT system scans the sample and the axial displacement in the layer is detected at each spatial location, where the smallest detectable displacement in the layer is determined by the axial resolution of the OCT system (5.5 µm)^38^. Stress can then be inferred from the measured OCT data and the pre-characterised stress–strain curve of the compliant layer.

### Silicone phantom fabrication

Four tissue-mimicking silicone inclusion phantoms are used as test targets for both CBOP and OCT-based optical palpation. The phantoms are cylindrical with a diameter of 15 mm and a height of 2 mm. The soft base of the phantoms are fabricated from a two-part silicone elastomer (Elastosil P7676, Wacker Chemie, Munich, Germany) which has an elasticity of 20 kPa at 10% strain, using procedures described previously^46^. Four inclusions with sizes of 5 × 5 × 1 mm, 2.5 × 2.5 × 1 mm, 1 × 1 × 1 mm and 0.5 × 0.5 × 1 mm were fabricated from a silicone elastomer with an elasticity of 160 kPa at 10% strain (Elastosil RT601, Wacker Chemie, Munich, Germany) and embedded 500 µm below the surface of the phantom. The base and inclusions of each phantom contain 0.25 mg/ml and 2 mg/ml of TiO_2_ powder, respectively, to provide optical contrast for OCT imaging.

### Clinical protocol

Freshly excised human breast samples from two mastectomy surgeries were imaged in this study. After surgery, these samples were immediately transferred from the operating theatre to the pathology department at Fiona Stanley Hospital, Western Australia. A pathologist dissected the sample to extract a ~ 3 × 2 × 1 cm sample which was then imaged using CBOP. During imaging, the specimen was kept hydrated in saline.

Following imaging, the sample was bisected and placed into two cassettes and a pathologist applied ink to the edges of the specimen to mark the orientation. The sample was then fixed in 10% neutral-buffered formalin and embedded in paraffin following standard histopathological protocols. The paraffin block was sectioned in the same reference plane as optical palpograms and stained with haematoxylin and eosin. This study was approved by the Sir Charles Gairdner and Osborne Park Health Care Group Human Research Ethics Committee (HREC No: 2007-152) and the Fiona Stanley Hospital Research Governance Office (FSH-2015-032), and informed consent was obtained from the patients prior to surgery. All methods were performed in accordance with the relevant guidelines and regulations, including following good clinical practices described at the International Conference on Harmonisation.

## Results

### Inclusion phantoms

**Figure 4:**
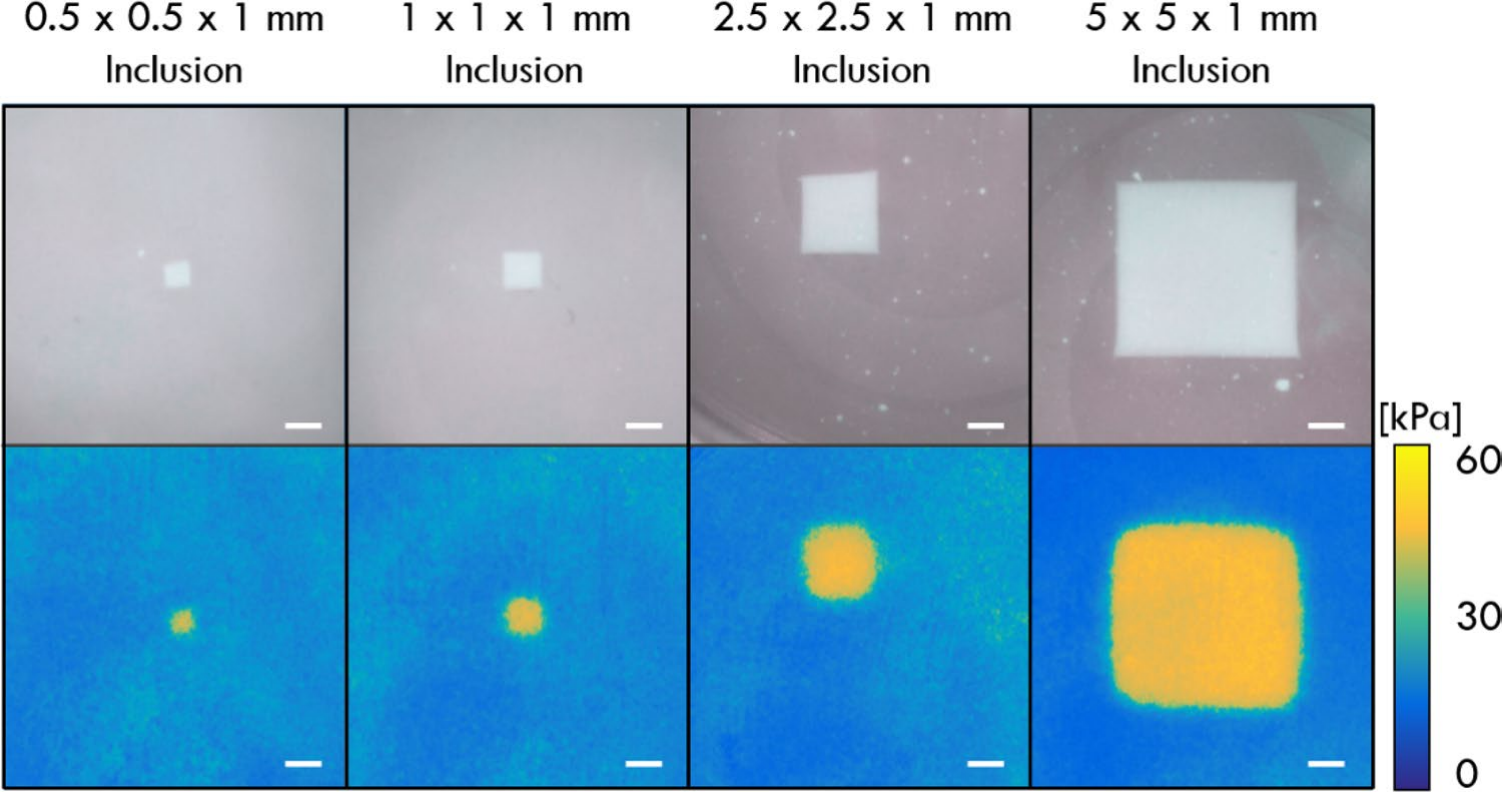
Photographs and optical palpograms acquired at 50% preloaded strain for four different-sized inclusion phantoms. Photographs are provided for validation purposes only to show the relative positions of the inclusions and were taken without the micro-porous layer. Scale bars: 1 mm.

To validate our technique, we first tested it on the four structured silicone phantoms described in the last section. During imaging, compression was increased in steps of 10%, causing incremental strain on the micro-porous layers, thus increasing the optical transmission and changing the colour saturation detected by the camera. As the inclusion is much stiffer than the surrounding base material, the strain in the micro-porous layer above the inclusion is larger than that above the base, creating contrast in colour saturation and, subsequently, in optical palpograms. Figure 4 shows the photographs and optical palpograms obtained using CBOP for each of the four inclusion phantoms, at 50% preloaded strain. The optical palpograms were obtained using the method described in the last section, while the photographs were acquired without the micro-porous layer and the green layer. In the optical palpograms, the inclusions are clearly distinguished from the base material due to the higher stress in these regions, and the inclusion sizes are comparable to the true values delineated in the photographs. The average stress measured above the four inclusions was 46.5 ± 1.9 kPa, compared to 12.9 ± 0.8 kPa for the soft base, demonstrating a high mechanical contrast between the two materials. Note that in Fig. 4 the corners of the inclusions in the optical palpograms are not as sharp as in the photographs, due to the non-uniaxial stress distribution in the micro-porous layer at the regions above the inclusion corners.

To characterise the optical palpograms generated from CBOP, we compared the phantom results acquired using CBOP with those acquired using OCT-based optical palpation. In particular, we measured the contrast-to-noise ratio (CNR) and lateral resolution in the phantoms using both techniques. Similar to the definition described in previous work^59^, we define CNR as:

4 $$CNR=\frac{\left|{\mu }_{inc}-{\mu }_{base}\right|}{\sqrt{{{\sigma }_{inc}}^{2}+{{\sigma }_{base}}^{2}}} ,$$

where ${\mu }_{inc}$ and ${\mu }_{base}$ are the mean stress values taken from the inclusion and base respectively, and ${\sigma }_{inc}$ and ${\sigma }_{base}$ are the corresponding standard deviations. These metrics were computed from 200 × 200 µm regions taken at the centre of the inclusions and at the edge of the base material in the field-of-view. This measurement of CNR takes into account the noise over both the inclusion and base, which is essential when measuring materials with non-linear mechanical properties as the noise increases at higher strains. Optimal CNR for CBOP was measured at 50% preloaded strain, where it yielded comparable CNR to OCT-based optical palpation. An average CNR of 35.2 ± 6.5 was measured across the four inclusion phantoms for CBOP, compared to 33.0 ± 9.3 for OCT-based optical palpation. Importantly, this result demonstrates that using the much simpler optical imaging system in CBOP did not substantially degrade image contrast.

**Figure 5:**
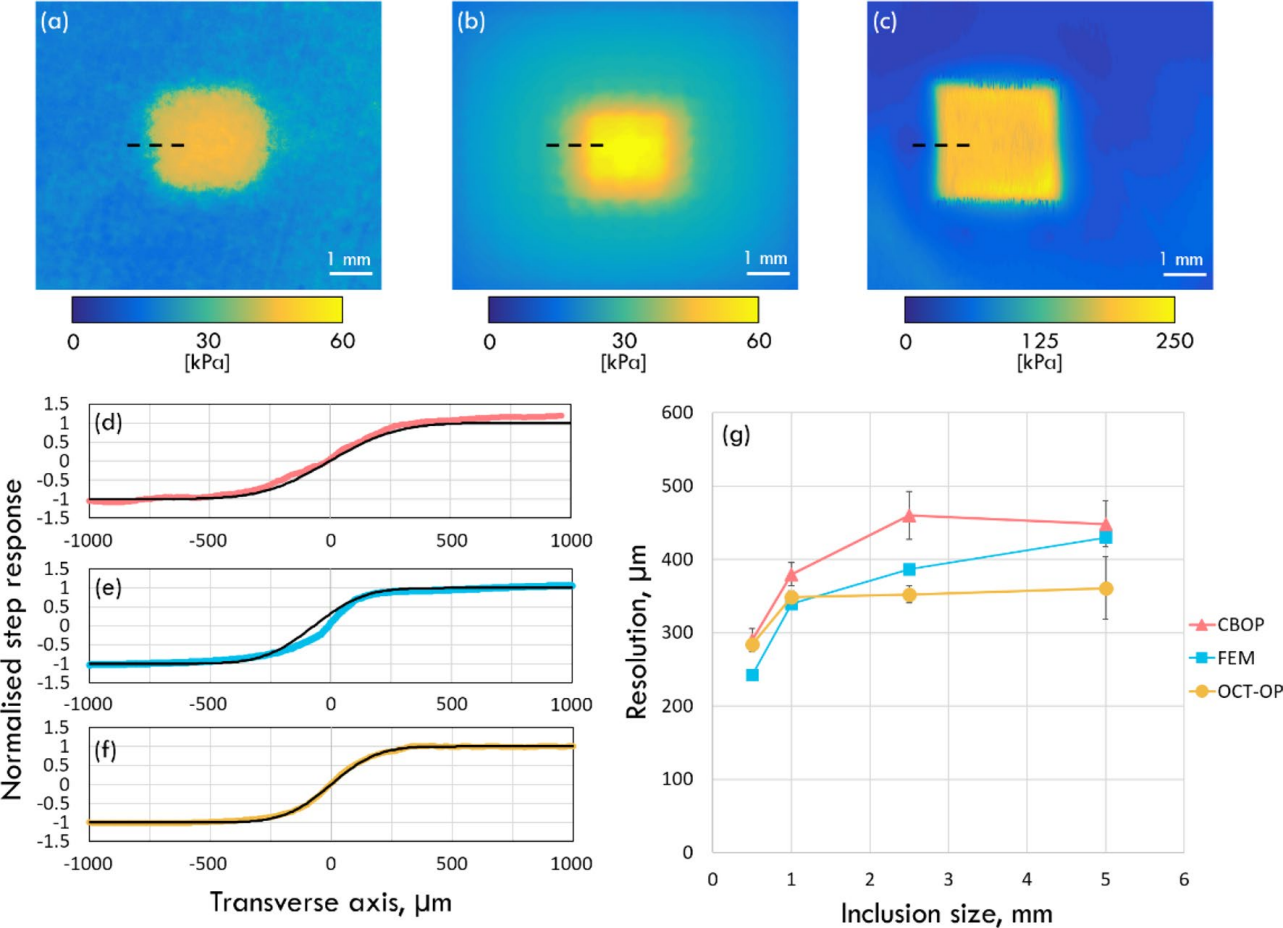
Analysis of the lateral resolutions of CBOP and OCT-based optical palpation. Optical palpograms acquired using (**a**) CBOP at 50% bulk preloaded strain, (**b**) FEM of CBOP and (**c**) OCT-based optical palpation at 50% bulk preloaded strain on a 2.5 × 2.5 × 1 mm inclusion embedded within a soft phantom. The normalised step response (coloured points) and error function (black line) of (**d**) CBOP, (**e**) FEM and (**f**) OCT-based optical palpation are taken across the boundary of the same inclusion phantom. (**g**) The lateral resolution measured using CBOP, OCT-based optical palpation and FEA, across five locations on each inclusion phantom with error bars representing one standard deviation.

Figure 5 shows the optical palpograms taken at 50% preloaded strain for the 2.5 × 2.5 × 1 mm inclusion phantom using CBOP (Fig. 5a), FEM (Fig. 5b) and OCT-based optical palpation (Fig. 5c). The lateral resolution of each technique was determined by measuring the step-response in stress measurements across the boundary between the inclusion and base, represented in Figs. 5d–f by the red (CBOP), blue (FEM) and yellow (OCT-based optical palpation) dots. An error function, represented by the black lines in Figs. 5d–f, was fitted to the stress measurements and the lateral resolution was defined as the 10–90% rise-distance of the error function. This procedure has previously been used to measure lateral resolution in both OCT^62^ and OCE^63^. The lateral resolution was measured at five different locations in each inclusion phantom and averaged to a mean value, as shown in Fig. 5g, where each error bar represents one standard deviation of the five measurements. CBOP demonstrated a lateral resolution of 290 µm for the smallest inclusion phantom, which increases with increasing inclusion size as shown in Fig. 5g. This trend of increasing lateral resolution along with increasing inclusion size is consistent with previous work in OCE^63^. The lateral resolution measured from FEM follows the same trend as the experimentally obtained results, albeit generally exhibiting a higher resolution. The lateral resolution calculated from FEM of the four inclusion phantoms is 430 µm, 390 µm, 340 µm and 240 µm in order of descending feature size with an average R^2^ value of 0.994. In Fig. 5a,b, the stress values of the base and inclusion measured from the experiment of CBOP correspond to 82.8% and 77.9%, respectively, of that measured in FEM. The differences between the lateral resolution measured in experiment and simulation are likely caused by the different friction conditions. In the experiment, the lubricant PDMS oil was likely squeezed out due to the compression, increasing the friction coefficient, while in the simulation, the friction coefficient was set as a constant, resulting in more lateral expansion and higher strain in the FEM which corresponds to higher stress values. To more accurately estimate the friction coefficient in the FEM, a more thorough measurement is required to characterise the change of friction coefficient in the experiment.

The OCT-based optical palpation measurements provide a baseline for CBOP results to be compared against. As illustrated in Fig. 5g, the measured lateral resolution from OCT-based optical palpation is 280 µm for the smallest inclusion, similar to that measured using CBOP. In both methods, the lateral resolution degrades as the inclusion size increases, until reaching a limit. This trend has been reported previously^63^ and is attributed to the restriction on lateral deformation of the base, imposed by the friction between the imaging window and stress layer. As inclusion size increases, the lateral deformation of the base situated between the inclusion and imaging window becomes increasingly restricted, resulting in a flattened strain gradient across the boundary between the inclusion and base thus degrading the lateral resolution. Above a certain inclusion size, the effect of the friction on the strain gradient across the boundary is unchanged, resulting in a limit on the lateral resolution. From Fig. 5g, it is observed that the lateral resolution in OCT-based optical palpation reaches this limit before that of CBOP as friction is more pronounced in thinner layers^64^ (the layers used in OCT-based palpation were 500 µm thick compared to 1.2 mm thick layers used in CBOP). In addition, CBOP generally has a lower resolution than OCT-based optical palpation, as the combined thickness of the green layer and the micro-porous layer effectively increase the depth of the inclusion. This effect of degraded resolution while imaging features at a greater depth has previously been reported for OCT-based optical palpation techniques, where optical palpation in general is able to detect mechanical contrast at a depth of 4–5 mm, provided sufficient compression has been applied to deform the sample above these features^38^. Beyond this depth, the assumption of uniaxial stress begins to fail and the stress distribution is no longer localised, making it challenging to generate optical palpograms of the sample features.

Note that the inclusion size in Fig. 5c appears larger than that in Figs. 5a,b, due to more lateral expansion of the inclusion in Fig. 5c. In the experiment, CBOP used a softer micro-porous layer, where much of the applied strain occurred in the layer, while OCT-based optical palpation used a thinner and stiffer stress layer, made from the same material as the base, resulting in the sample experiencing a higher strain for the same applied preloaded strain. The difference between the sample strains in the two methods is indicated by the higher stress values in Fig. 5c than those in Fig. 5a,b.

### Ex vivo human mastectomy samples

**Figure 6:**
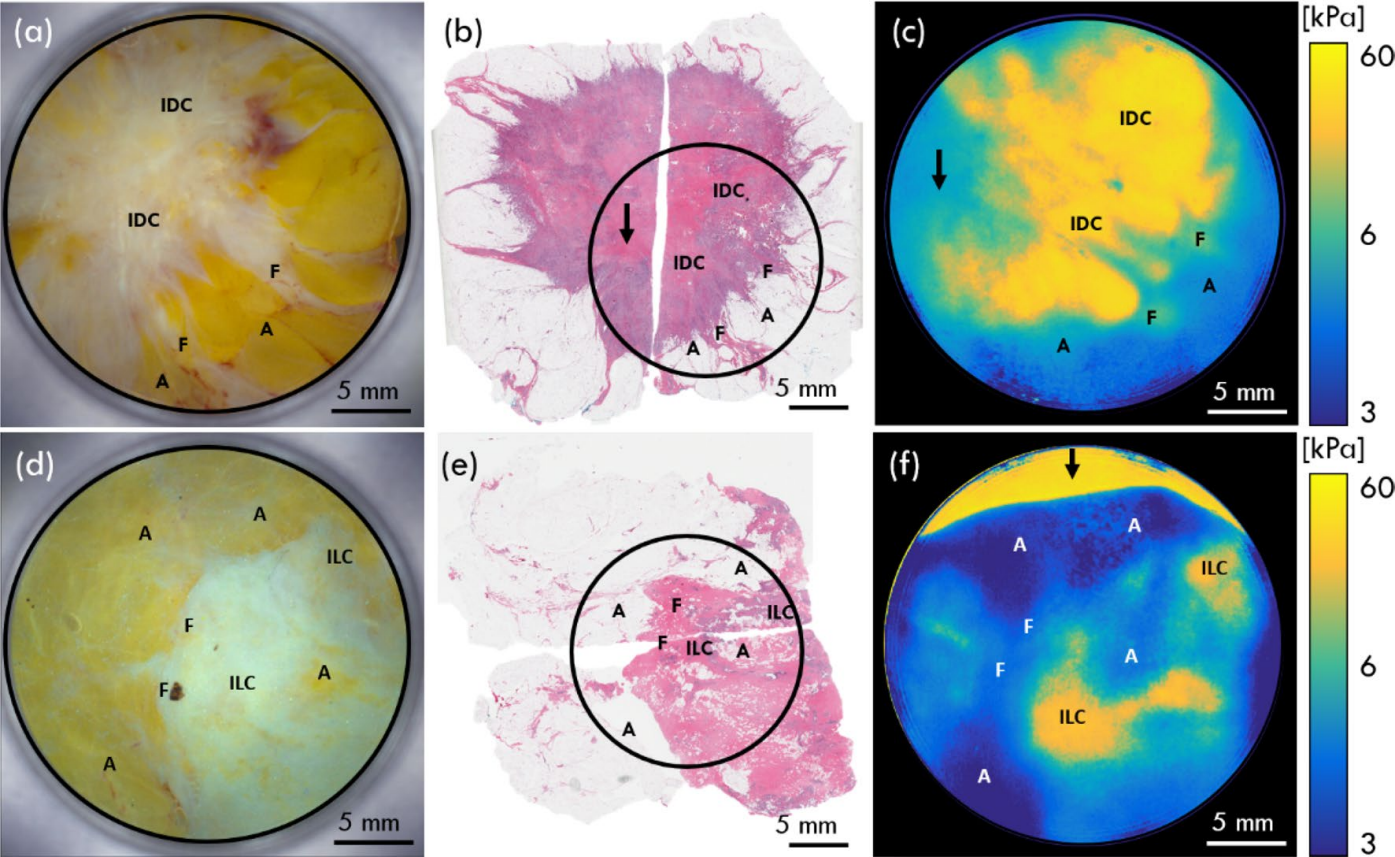
CBOP performed on two freshly excised mastectomy specimens containing (**a**–**c**) IDC and (**d**–**f**) ILC. (**a**) Photograph, (**b**) histology and (**c**) optical palpogram at 30% preloaded strain. (**d**) Photograph (**e**) histology image and (**f**) optical palpogram at 60% preloaded strain. The images have been annotated to show regions of invasive ductal carcinoma (IDC), invasive lobular carcinoma (ILC), fibrous tissue (F) and adipose tissue (A) and black circles on the histology images mark the approximate region where CBOP was taken relative to the whole specimen. The arrows in (**b**,**c**,**f**) indicate regions of imaging artefacts. Optical palpograms have been displayed on a logarithmic scale to enhance mechanical contrast between tissue types.

To validate the performance of CBOP on human tissue, we imaged freshly excised sections of human breast tissue comprising of tumour and adipose, from mastectomy specimens obtained from patients at Fiona Stanley Hospital, Western Australia. In Fig. 6, we compare the photograph, the histology image and the optical palpogram acquired from CBOP. With the aid of expert advice from the pathologists involved in the study, labelling of features in the histology images provided a means to validate contrast obtained in the co-registered optical palpograms.

The first scan was acquired on a specimen from a 59-year-old patient. As shown in Fig. 6a, a photograph of the tissue specimen was acquired after performing CBOP on the same tissue location. The corresponding histology image is presented in Fig. 6b which was acquired at a depth of ~ 100 µm below the tissue surface and is marked with a black circle to denote the CBOP scanning area. Pathologists identified the presence of invasive ductal carcinoma (IDC), fibrous tissue (F) and adipose tissue (A) in the histology image, and these regions have been annotated accordingly on Fig. 6a–c. The optical palpogram, shown in Fig. 6c, was acquired at 30% preloaded strain and exhibits a region of elevated stress at the centre of the image, corresponding to invasive ductal carcinoma (IDC) from the histology image. This suggests that IDC is stiffer than surrounding benign tissues, which is consistent with previous results acquired using QME^21,22,41^. Similarly, regions of moderate and low stress match up well with fibrous tissue and adipose tissue detected in the histology image. It is worth highlighting here that while the regions in the histology image correspond with those in the optical palpograms, the imaged area is slightly increased due to the lateral expansion of the tissue under compression during CBOP, when compared to the histology image. Note that there is a region of lower stress in Fig. 6c, marked by a black arrow, which appears to be either fibrous tissue or adipose tissue, however, this is likely to be an artefact caused by variation in the sample thickness, leading to an underestimation of stress. The same region is highlighted by a black arrow in histology Fig. 6b where IDC was not annotated due to the variation in sample thickness.

The second specimen, taken from a 69-year-old patient, is presented in Figs. 6d–f. Figure 6d shows a photograph acquired after performing CBOP and Fig. 6e is the histology image of the specimen acquired at ~ 100 µm below the tissue surface, with a black circle highlighting the same region of the field-of-view of CBOP and photograph. Figure 6f is the optical palpogram of the specimen acquired using CBOP. Due to different tissue geometries and mechanical properties, the optimal preloaded strain can vary between samples. For this specimen, CBOP was performed at 60% preloaded strain, which ensures high contrast between the different tissue types present. Postoperative histology of the specimen (Fig. 6e) revealed the presence of invasive lobular carcinoma (ILC), fibrous tissue (F) and adipose tissue (A). Once again, annotation was performed by a pathologist and the regions of ILC in the histology image correspond well with the regions of elevated stress in the optical palpogram (Fig. 6f), which is consistent with previous studies of the mechanical properties of ILC^22,65^. In Fig. 6f, an arrow denotes an imaging artefact at the top of the optical palpogram that appears as a region of high stress. This artefact was produced when a portion of the green layer was imaged without the micro-porous layer covering it, giving the appearance of high stress.

## Discussion

In this paper, we demonstrate CBOP, a new optical elastography platform, capable of generating 2-D maps of surface stress by using a digital camera and a micro-porous silicone layer. A previous digital-camera-based technique has been proposed in optical elastography by directly imaging the tissue as it is subjected to a tensile loading^66^. Whilst this approach can generate mechanical contrast in tissue, it has the limitation that it relies on visualising tissue features to map deformation. As such, in tissue regions where the contrast is low in the optical image, elastography measurements are not possible. In addition, in many cases, tensile loading is either not easily implemented or undesirable. Our approach with CBOP overcomes these issues by generating contrast from light transmitted through the micro-porous layer and this method is independent of the visual contrast of the tissue. CBOP represents a simple and easy-to-use approach to optical elastography, making it more accessible to a broader range of applications. Furthermore, as the data is acquired from several digital photographs, it greatly simplifies the data processing, readily providing high-speed imaging, which is vital in time-sensitive clinical applications.

In CBOP, we often compress the sample and layers to ~ 50% preloaded strain to optimise CNR. The issue with this is that the compressive force required to achieve such a high strain results in the lubricant PDMS oil being forced out, leading to an increase in friction^67^ and a degradation in lateral resolution, as described in “Results”. This issue can be resolved by using a softer material for the substrate of the micro-porous layer, which allows optical palpograms to be acquired at lower preloaded strains. With this approach, we can minimise the effect of friction, thus improving the lateral resolution.

An advantage of CBOP, in common with optical palpation in general, is that the layer complies to the tissue surface to provide a relatively uniform stress distribution at the tissue surface. However, when tissue samples exhibit large variation in surface topography, the layer is not able to fully comply with the sample^68^. While this issue is present in all optical palpation techniques, it is more evident in CBOP than in the OCT-based technique as the micro-porous layer is compressible, meaning it exhibits a lower Poisson’s ratio, and is therefore, less likely to conform to the tissue surface. This may lead to non-uniform contact and can lower the contrast between tumour and benign tissue, as seen in Fig. 6c. To achieve more even contact with the tissue sample, the micro-porous layer needs to be thick enough to conform to the surface of the tissue, with some compromise in lateral resolution. Alternatively, uniform contact could be achieved by reducing the elasticity of the homogeneous green layer, allowing it to better conform to the topography of the tissue. In future studies, this improvement may increase the accuracy of CBOP in identifying malignant and benign tissues.

Here we have used CBOP to image breast tissue, a heterogeneous tissue which displays nonlinear mechanical properties. Due to this nonlinearity, the different local strain experienced by the sample during CBOP will have a strong effect on the apparent stiffness of the individual tissue constituents, potentially reducing the contrast between soft adipose tissue and stiff tumour. One solution proposed for use in OCE synthesizes a map of stiffness by reassembling A-scans taken from a series of OCE images under increasing load, such that the stress in the layer is uniform over the entire B-scan^68^. Implementation of this type of standardisation in CBOP, however, may prove challenging as CBOP is only able to visualise the strain in the layer rather than the strain in the sample. As CBOP is a qualitative technique, the absolute value of elasticity is less relevant, provided that regions of high stress reliably identifies tumour. Whilst there is scope to explore this further, a previous study using OCT-based optical palpation on 34 freshly excised human breast tissue samples indicates that the effect of nonlinearity is not large enough to obscure the ability to localise tumour^40^.

The drawback of CBOP is that it can only generate 2-D optical palpograms, which are qualitative measurements of tissue mechanical properties. In clinical applications, such as tumour margin assessment, detailed knowledge of not only the stress, but also elasticity, which is a quantitative measurement of the mechanical properties of tissue, will likely be required to remove subjectivity and variability between measurements. In addition, the information of the depth of certain features is critical in guiding clinicians to suspicious regions of tissue. To provide 3-D quantitative measurements of mechanical properties, inverse methods can be used, as has been proposed in tactile imaging applications^69–71^, to generate 3-D elastography with higher resolution and accuracy.

To date, optical palpation has been developed primarily for clinical applications, typically suited to advanced healthcare systems, owing to the high cost of OCT. A benefit of CBOP is that it offers a cost-effective implementation of optical palpation, which can broaden the applications of optical palpation, particularly in remote and low-resource settings. For example, CBOP may aid in assessment of the cavity wall following a wide local excision in breast cancer surgery, where other intraoperative techniques would prove too costly for low-resource healthcare^72,73^. In addition, CBOP may be suitable for remote applications beyond medicine, such as agriculture, where the bulk stiffness of plant leaves is an indicator of plant health^74^.

In this proof-of-principle study, CBOP was performed using a benchtop design, with a high-resolution CMOS camera to ensure high quality optical palpograms. In future work, this technique can be implemented using more cost-effective CCD/CMOS cameras. As CBOP consists of only a digital camera and the accompanying layers, it can be readily incorporated in a smartphone, with a broad range of potential applications, e.g. the detection of malignant skin lesions^75,76^. While there is already a great deal of research into the use of smartphones in diagnostic applications, augmenting these techniques with elastography holds potential for increasing diagnostic accuracy. In addition, the benchtop scanning system is restricted to *ex vivo* studies, whilst development of a small form-factor probe would permit scanning of *in vivo* tissue intraoperatively, e.g. the assessment and localisation of hepatic tumours, which present as stiff lesions, during liver surgery^65^. Furthermore, the use of a digital camera allows for the design of a wireless probe, which is preferable in a robust clinical setting due to the dexterity and freedom of motion it offers.

## Conclusion

In this paper, we have presented CBOP, a cost-effective optical elastography platform capable of mapping stress at the tissue surface. CBOP has demonstrated a resolution of 290 µm and a CNR of 35.2, similar to values obtained with OCT-based optical palpation. In addition, we have demonstrated that CBOP can detect the mechanical contrast between invasive tumour and benign tissue in excised human breast specimens. This technique can be readily developed into a small form factor handheld probe due to the use of a simple digital camera, enhancing the potential for clinical translation.

## Data Availability

The data in this work is available upon request.

## References

[1] Kennedy BF, Wijesinghe P, Sampson DD (2017). The emergence of optical elastography in biomedicine. Nat. Photonics.

[2] Wang S, Larin KV (2015). Optical coherence elastography for tissue characterization: A review. J. Biophotonics.

[3] Kennedy BF, Kennedy KM, Sampson DD (2014). A review of optical coherence elastography: Fundamentals, techniques and prospects. IEEE J. Sel. Top. Quant..

[4] Liang X, Crecea V, Boppart SA (2010). Dynamic optical coherence elastography: A review. J. Innov. Opt. Heal. Sci..

[5] Zaitsev, V. Y. *et al.* Strain and elasticity imaging in compression optical coherence elastography: the two-decade perspective and recent advances. *J. Biophotonics***n/a**, e202000257. 10.1002/jbio.202000257 32749033

[6] Fung YC (2013). Biomechanics: Mechanical Properties of Living Tissues.

[7] Plodinec M (2012). The nanomechanical signature of breast cancer. Nat. Nanotechnol..

[8] Ophir J (1996). Elastography: Ultrasonic imaging of tissue strain and elastic modulus in vivo. Ultraschall Med..

[9] Greenleaf JF, Fatemi M, Insana M (2003). Selected methods for imaging elastic properties of biological tissues. Annu. Rev. Biomed. Eng..

[10] Li QS, Lee GYH, Ong CN, Lim CT (2008). AFM indentation study of breast cancer cells. Biochem. Bioph. Res. Co..

[11] Righetti R, Srinivasan S, Ophir J (2003). Lateral resolution in elastography. Ultrasound Med. Biol..

[12] Manduca A (2001). Magnetic resonance elastography: Non-invasive mapping of tissue elasticity. Med. Image Anal..

[13] Sanderson RW, Curatolo A, Wijesinghe P, Chin L, Kennedy BF (2019). Finger-mounted quantitative micro-elastography. Biomed Opt. Express.

[14] Kennedy KM, Kennedy BF, McLaughlin RA, Sampson DD (2012). Needle optical coherence elastography for tissue boundary detection. Opt. Lett..

[15] van Soest G, Mastik F, de Jong N, van der Steen AFW (2007). Robust intravascular optical coherence elastography by line correlations. Phys. Med. Biol..

[16] Schmitt JM (1998). OCT elastography: Imaging microscopic deformation and strain of tissue. Opt. Express.

[17] Liang X, Boppart SA (2010). Biomechanical properties of in vivo human skin from dynamic optical coherence elastography. IEEE T. Bio-med. Eng..

[18] Sovetsky AA, Matveyev AL, Matveev LA, Shabanov DV, Zaitsev VY (2018). Manually-operated compressional optical coherence elastography with effective aperiodic averaging: Demonstrations for corneal and cartilaginous tissues. Laser Phys. Lett..

[19] Plekhanov AA (2020). Histological validation of in vivo assessment of cancer tissue inhomogeneity and automated morphological segmentation enabled by Optical Coherence Elastography. Sci. Rep..

[20] Larin KV, Sampson DD (2017). Optical coherence elastography—OCT at work in tissue biomechanics [Invited]. Biomed. Opt. Express.

[21] Allen WM (2018). Wide-field quantitative micro-elastography of human breast tissue. Biomed. Opt. Express.

[22] Kennedy KM (2020). Diagnostic accuracy of quantitative micro-elastography for margin assessment in breast-conserving surgery. Cancer Res..

[23] Kennedy BF (2015). Investigation of optical coherence microelastography as a method to visualize cancers in human breast tissue. Cancer Res..

[24] Kirby MA (2017). Optical coherence elastography in ophthalmology. J. Biomed. Opt..

[25] Wang S (2018). Biomechanical assessment of myocardial infarction using optical coherence elastography. Biomed. Opt. Express.

[26] Ford MR (2011). Method for optical coherence elastography of the cornea. J. Biomed. Opt..

[27] Koski KJ, Yarger JL (2005). Brillouin imaging. Appl. Phys. Lett..

[28] Scarcelli G, Yun SH (2008). Confocal Brillouin microscopy for three-dimensional mechanical imaging. Nat. Photonics.

[29] Prevedel R, Diz-Muñoz A, Ruocco G, Antonacci G (2019). Brillouin microscopy: An emerging tool for mechanobiology. Nat. Methods.

[30] Margueritat J (2019). High-frequency mechanical properties of tumors measured by brillouin light scattering. Phys. Rev. Lett..

[31] Scarcelli G, Pineda R, Yun SH (2012). Brillouin optical microscopy for corneal biomechanics. Invest. Ophth. Vis. Sci..

[32] Cherfan D (2013). Collagen cross-linking using rose bengal and green light to increase corneal stiffness. Invest. Ophth. Vis. Sci..

[33] Scarcelli G (2015). Noncontact three-dimensional mapping of intracellular hydromechanical properties by Brillouin microscopy. Nat. Methods.

[34] Curatolo A (2016). Ultrahigh-resolution optical coherence elastography. Opt. Lett..

[35] Hepburn MS (2020). Three-dimensional imaging of cell and extracellular matrix elasticity using quantitative micro-elastography. Biomed. Opt. Express.

[36] Kress-Rogers E, Brimelow CJB (2001). Instrumentation and Sensors for the Food Industry.

[37] Tiwana MI, Redmond SJ, Lovell NH (2012). A review of tactile sensing technologies with applications in biomedical engineering. Sensor. Actuat. A-Phys..

[38] Kennedy KM (2014). Optical palpation: Optical coherence tomography-based tactile imaging using a compliant sensor. Opt. Lett..

[39] Eshaghian S (2015). Optical palpation in vivo: Imaging human skin lesions using mechanical contrast. J. Biomed. Opt..

[40] Allen WM (2019). Optical palpation for the visualization of tumor in human breast tissue. J. Biophotonics.

[41] Kennedy KM (2015). Quantitative micro-elastography: Imaging of tissue elasticity using compression optical coherence elastography. Sci. Rep..

[42] Zaitsev VY, Dyskin A, Pasternak E, Matveev L (2009). Microstructure-induced giant elastic nonlinearity of threshold origin: Mechanism and experimental demonstration. Europhys. Lett..

[43] Kobayashi T, Saitoh H, Fujii N, Hoshino Y, Takanashi M (1993). Porous membrane of polydimethylsiloxane by hydrosilylation cure: Characteristics of membranes having pores formed by hydrogen foams. J. Appl. Polym. Sci..

[44] Choi S-J (2011). A polydimethylsiloxane (PDMS) sponge for the selective absorption of oil from water. ACS Appl. Mater. Inter..

[45] Zhu D, Handschuh-Wang S, Zhou X (2017). Recent progress in fabrication and application of polydimethylsiloxane sponges. J. Mater. Chem. A.

[46] Lamouche G (2012). Review of tissue simulating phantoms with controllable optical, mechanical and structural properties for use in optical coherence tomography. Biomed. Opt. Express.

[47] Larsson M, Hill A, Duffy J (2012). Suspension stability; why particle size, zeta potential and rheology are important. Ann. T. Nord. Rheol. Soc..

[48] Durian DJ, Weitz DA, Pine DJ (1991). Multiple light-scattering probes of foam structure and dynamics. Science.

[49] Bohren CF, Huffman DR (2008). Absorption and Scattering of Light by Small Particles.

[50] Deng X, Gan X, Gu M (2003). Monte Carlo simulation of multiphoton fluorescence microscopic imaging through inhomogeneous tissuelike turbid media. J. Biomed. Opt..

[51] van de Hulst HC (1981). Light Scattering by Small Particles.

[52] Cox AJ, DeWeerd AJ, Linden J (2002). An experiment to measure Mie and Rayleigh total scattering cross sections. Am. J. Phys..

[53] Borovinšek M, Vesenjak M, Higa Y, Shimojima K, Ren Z (2019). Characterization of geometrical changes of spherical advanced pore morphology (APM) foam elements during compressive deformation. Materials.

[54] Smith RA, Paulus MJ, Branning JM, PhillipsS PJ (2001). X-ray computed tomography on a cellular polysiloxane under compression. J. Cell. Plast..

[55] Tscharnuter D, Jerabek M, Major Z, Lang RW (2011). Time-dependent poisson’s ratio of polypropylene compounds for various strain histories. Mech. Time-Depend. Mat..

[56] Wang B, Krause S (1987). Properties of dimethylsiloxane microphases in phase-separated dimethylsiloxane block copolymers. Macromolecules.

[57] Smith, A. R. Color gamut transform pairs. in *Proceedings of the 5th annual conference on Computer graphics and interactive techniques - SIGGRAPH ’78* 12–19 (ACM Press, 1978). 10.1145/800248.807361.

[58] Wijesinghe P, Sampson DD, Kennedy BF (2017). Computational optical palpation: A finite-element approach to micro-scale tactile imaging using a compliant sensor. J. R. Soc. Interface.

[59] Mooney M (1940). A theory of large elastic deformation. J. Appl. Phys..

[60] Rivlin RS, Rideal EK (1948). Large elastic deformations of isotropic materials. IV. Further developments of the general theory. Philos. T. R. Soc. Lond..

[61] Papazafeiropoulos G, Muñiz-Calvente M, Martínez-Pañeda E (2017). Abaqus2Matlab: A suitable tool for finite element post-processing. Adv. Eng. Softw..

[62] Curatolo A, Kennedy BF, Sampson DD (2011). Structured three-dimensional optical phantom for optical coherence tomography. Opt. Express.

[63] Hepburn MS, Wijesinghe P, Chin L, Kennedy BF (2019). Analysis of spatial resolution in phase-sensitive compression optical coherence elastography. Biomed. Opt. Express.

[64] Kuhn H (2000). Uniaxial compression testing. Mechanical Testing and Evaluation.

[65] McKnight AL (2002). MR elastography of breast cancer: Preliminary results. Am. J. Roentgenol..

[66] Zhang Y, Brodell RT, Mostow EN, Vinyard CJ, Marie H (2009). In vivo skin elastography with high-definition optical videos. Skin Res. Technol..

[67] Carbone G, Persson BNJ (2004). Dewetting at soft viscoelastic interfaces. J. Chem. Phys..

[68] Sovetsky AA (2020). Full-optical method of local stress standardization to exclude nonlinearity-related ambiguity of elasticity estimation in compressional optical coherence elastography. Laser Phys. Lett..

[69] Wellman, P. S. *et al.* Tactile imaging: a method for documenting breast masses. in *Proceedings of the First Joint BMES/EMBS Conference. 1999 IEEE Engineering in Medicine and Biology 21st Annual Conference and the 1999 Annual Fall Meeting of the Biomedical Engineering Society (Cat. N* vol. 2 1131 vols2- (1999).

[70] Hosseini SM, Amiri M, Najarian S, Dargahi J (2007). Application of artificial neural networks for the estimation of tumour characteristics in biological tissues. Int. J. Med. Robot. Comp..

[71] Lee J-H, Won C-H (2013). The tactile sensation imaging system for embedded lesion characterization. IEEE J. Biomed. Health.

[72] Osborn JB, Keeney GL, Jakub JW, Degnim AC, Boughey JC (2011). Cost-effectiveness analysis of routine frozen-section analysis of breast margins compared with reoperation for positive margins. Ann. Surg. Oncol..

[73] James TA (2009). Intraoperative ultrasound versus mammographic needle localization for ductal carcinoma in situ. Ann. Surg. Oncol..

[74] Touchette BW, Marcus SE, Adams EC (2014). Bulk elastic moduli and solute potentials in leaves of freshwater, coastal and marine hydrophytes. Are marine plants more rigid?. AoB Plants.

[75] Esteva A (2017). Dermatologist-level classification of skin cancer with deep neural networks. Nature.

[76] Chuchu N (2018). Smartphone applications for triaging adults with skin lesions that are suspicious for melanoma. Cochrane DB Syst. Rev..

